# “I was Confused … and Still am” Barriers Impacting the Help-Seeking Pathway for an Autism Diagnosis in Urban North India: A Mixed Methods Study

**DOI:** 10.1007/s10803-021-05047-z

**Published:** 2021-05-20

**Authors:** Supriya Bhavnani, Georgia Lockwood Estrin, Rashi Arora, Divya Kumar, Minal Kakra, Vivek Vajaratkar, Monica Juneja, Sheffali Gulati, Vikram Patel, Jonathan Green, Gauri Divan

**Affiliations:** 1 Child Development Group, Sangath, House 451 Bhatkar Waddo, Succor, Bardez, Goa 403501 India; 2 Centre for Chronic Conditions and Injuries, Public Health Foundation of India, Plot No. 47, Sector 44, Institutional Area, Gurgaon, 122002 India; 3 Centre for Brain and Cognitive Development, Birkbeck, University of London, Malet Street, WC1E 7HX London, UK; 4 Department of Orthopaedic Surgery, Goa Medical College, Bambolim, Goa 403202 India; 5 Maulana Azad Medical College, Balmiki Basti, New Delhi, India 110002; 6 Child Neurology Division, Department of Pediatrics, All India Institute of Medical Sciences, Ansari Nagar, New Delhi, 110029 India; 7 Department of Global Health and Social Medicine, Harvard Medical School, 641 Huntington Ave, Boston, MA 02115 USA; 8 Department of Global Health and Population, Harvard T H Chan School of Public Health, 655 Huntington Ave, Boston, MA 02115 USA; 9 Royal Manchester Children’s Hospital, University of Manchester and Manchester Academic Health Sciences Centre, Oxford Rd, Manchester, M13 9PL UK

**Keywords:** Autism Spectrum Disorders, Help-seeking pathway, Diagnosis, Delay

## Abstract

Timely recognition of autism in children is integral to improve developmental outcomes. This study used mixed-methods (84 case-registers and 20 in-depth interviews with caregivers of children with a diagnosis of autism) to explore the extent to which the nature of parental concerns and prior knowledge of developmental disorders impact the time between symptom recognition and autism diagnosis, and the contextual family, societal and health-system related factors that impede the autism help-seeking pathway. Lack of awareness of age-appropriate child developmental milestones, apparent amongst the community and health professionals, contributed to a 1.5-year delay between parental concerns and autism diagnosis. Recommendations to shorten this help-seeking pathway include harnessing the potential of non-specialist workers to increase awareness and enable developmental monitoring of young children through scalable tools.

**Supplementary Information:**

The online version contains supplementary material available at 10.1007/s10803-021-05047-z.

Autism Spectrum Disorder (henceforth ‘autism’) is a complex neurodevelopmental condition that manifests early in childhood and is characterised by social communication and interaction impairments, restricted interests and increased repetitive behaviours (American Psychiatric Association [APA], 2013). It is estimated that the global prevalence of autism is 1 in 132 (Baxter et al., 2015) and a recent study conducted in India also found similar prevalence rates; that of 1% in 2–6-year old and 1.4% in 6–9-year old children (Arora et al., 2018). A study in 2018 estimated that there are 1.2 million children under 5-years age living with autism in South Asia, accounting for over a quarter of the global prevalence (Olusanya et al., 2018).

Randomised controlled trials conducted in the past few decades have successfully demonstrated that interventions implemented early in childhood have the potential to improve the social-communication and adaptive behaviour outcomes of children with a diagnosis of autism (Green & Garg, 2018; Landa, 2018). This is likely due to the high degree of plasticity of the brain in the first few years of life, when it is most amenable to change and adaptable to the environment (Estrin & Bhavnani, 2020). A crucial first step for implementing early interventions is the timely identification of autism. However, in most low and middle income countries (LAMIC), a large ‘detection gap’ exists wherein recognition of autism in children either does not occur at all, or at best occurs late in their development resulting in a tragic missed opportunity to improve developmental and behavioural outcomes (Dasgupta et al., 2016). This is partly because an autism diagnosis is dependent on detailed observation of child behaviour by child development specialists such as clinical psychologists and paediatricians; these services are scarcely available in low resource settings.

In most settings, including high income countries (HIC), the average age at which children obtain an autism diagnosis is approximately 4 years (Maenner, 2020). This is despite the fact that there seems to be emerging consensus that autism can be reliably and stably diagnosed in children around 2 years old (Guthrie et al., 2013; Lord et al., 2006; Ozonoff et al., 2015). Indeed, studies from both HIC and LAMIC have demonstrated that parents and caregivers often have concerns early in the child’s development, however there is a delay, sometimes of many years, till they obtain a diagnosis of autism (Daniels & Mandell, 2014).

In order to minimise this delay in diagnosis and the subsequent access to interventions, it is crucial to understand its underlying reasons. Both quantitative and qualitative approaches have been used to investigate the potential factors that contribute to delayed autism diagnosis in young children. Reports from HIC have pointed to demographic factors like ethnicity and socioeconomic status, as well as contextual factors like the response of health care providers to parental concerns (Zuckerman et al., 2015a, 2015b, 2017). These contextual factors are likely to differ in LAMIC which have challenges that are unique to each setting. In fact, existing literature, summarised into a framework through a recent systematic review, suggests that the recognition, interpretation and reporting of symptoms, all differ based on the cultural context of the family of the child with autism (Leeuw et al., 2020).

The last comprehensive exploration into the types of developmental and behavioural concerns that get recognised by parents, and the contextual family and societal factors that impede autism diagnosis in India was done a decade and a half ago, in 2005 (Daley, 2004). This study used qualitative methods to illustrate that delays in diagnosis can also be attributed to parental impressions of their child’s behaviour and environmental and cultural factors. A recent study from Odisha in India conducted in-depth interviews with parents of children with autism, but with only a limited focus on the contextual barriers to the help-seeking pathway for autism (Gupta et al., 2019; Mahapatra et al., 2019).

There is thus a need to build on the evidence base of the contextual barriers that prolong the help-seeking pathway followed by families of children with autism in India in order to identify opportunities that can facilitate the provision of an autism diagnosis and access to available interventions at a younger age.

In this study, we used a mixed-methods design to explore: (1) The extent to which the nature of parental concerns and prior knowledge of developmental disorders impact the time between symptom recognition and autism diagnosis, and (2) the contextual family, societal and health-system related factors that impede the autism help-seeking pathway. We triangulated our quantitative and qualitative findings to make recommendations of potential actions that can be taken at various stages within the help-seeking pathway to minimize its length.

## Methods

### Study Site and Participant Details

Participants for the quantitative component of the study were recruited from the Department of Paediatric Neurology at All India Institute of Medical Sciences (AIIMS) between July 2017 and February 2018. For the qualitative component, a distinct set of primary caregivers of 20 children with autism were recruited, 10 each from AIIMS and Maulana Azad Medical College and associated Lok Nayak Hospital (MAMC). Participants represent urban families seeking care in government run tertiary care centres in New Delhi, India. All children included in the analysis had received an autism diagnosis based on DSM-V (American Psychiatric Association [APA], 2013) criteria from clinicians at these hospitals. Potential participants were explained the purpose of the study at the end of their clinical appointment by clinicians at the two sites. If they expressed interest, they were given details through the participation information sheet which was followed up by a discussion with the research team. Potential participants were informed that their clinical care would not be impacted by their decision to participate in the study. Informed consent was obtained at the time of the interview.

### Study Procedures

A database maintained by the Paediatric Neurology Department at AIIMS, the recruitment site for the quantitative component of this study, comprises case-registers for every family that brings a child in for consultation. These case-registers include data on (a) demographic information, (b) clinician’s notes from an open-ended interview wherein the parent describes the symptoms of their child, henceforth referred to as ‘presenting concerns’, (c) presence of comorbid seizures and (d) the child’s score on the Childhood Autism Rating Scale (CARS) (Eric Schopler et al., 2010). Upon enrolment into this study, participant case-registers were accessed and the data described above was extracted and tabulated. In addition, during enrolment, parents were asked to recall the age of their child when they first had concerns about their development and when they obtained an autism diagnosis, henceforth referred to as ‘age at initial parental concern’ and ‘age at diagnosis’ respectively. Parents were also asked if they had heard of autism or any other developmental disorder before their own child was diagnosed—henceforth called ‘prior knowledge’, and included as an indicator in the quantitative analysis. A total of 90 case-registers were accessed; three were excluded due to missing data on age at initial parental concern and age at diagnosis and the CARS score of three children was found to be below 30—the recommended cut-off for autism diagnosis (Eric Schopler et al., 2010). The final sample for the quantitative component of the study comprised of 84 children.

For the qualitative component, semi-structured in-depth interviews were conducted in Hindi with parents or primary caregivers of 20 children with an autism diagnosis. Interviews ranged from 30 min to one-hour in duration. An interview guide was developed which included two areas of interest to this topic: (1) the nature of initial parental concerns and (2) parents’ experience of obtaining an autism diagnosis for their child including individuals consulted, the advice received and the action taken along each step of their help-seeking pathway. All interviewees consented for recording on a voice recorder; and expanded notation methodology (Halcomb & Davidson, 2006) was used to document the interview in English by a bilingual researcher. A subset of interviews were transcribed verbatim and translated to English, and all expanded notes and transcripts were checked for accuracy by bilingual researchers who read them while listening to the audio file simultaneously.

### Data Analysis

The quantitative data was analysed using Stata version 14 (*Stata: Software for Statistics and Data Science*, 2015). Age at initial parental concern, age at diagnosis and time to diagnosis (calculated as “age at diagnosis—age at initial parental concern”) were the dependent variables and these were treated as continuous. The independent variables were (a) sex of the child, (b) the type of presenting concerns documented from the case-registers and (c) parents’ revealing any *prior knowledge of developmental disorders*, including autism, before their child had been diagnosed.

The type of statistical test used to compare variables was based on their distributions (Nayak & Hazra, 2011). For normally distributed outcomes, t-tests were used to compare categorical independent variables, and Pearson’s correlation was used for continuous variables. For non-normal outcomes, Mann Whitney tests were used to compare categorical independent variables (Fisher’s exact test used if n ≤ 5 in any group), and Spearman’s correlation was used for continuous variables.

Transcripts and expanded notes from qualitative in-depth interviews were analysed using framework analysis, which has been widely used in health research, and especially to identify drivers and barriers to healthcare services (Gale et al., 2013). The aim of this analysis was to map a timeline on the pathway to care, to document how help is sought and advice received, from the initial recognition of developmental or behavioural concerns by parents, and at each step along the journey to a diagnosis, including the age at which a diagnosis was received (see Table 1 for detailed codebook and definitions). Following immersion in the interview transcripts, an initial codebook was developed. SB and GLE both independently coded all the data and reviewed each other’s codes while discussing each transcript. During a second immersion of the interviews, the codebook was further refined through discussion, and emerging themes were identified to form a finalised codebook, which was applied to the remaining interviews. Any divergences of coding between SB and GLE were discussed until consensus was reached.

**Table 1:** Codebook developed for framework analysis of qualitative data

Topics	Themes	Definitions
Initial recognition	Difficulties noticed for the first time	Kinds of difficulties or differenced noticed for the first time, e.g., speech problem, not playing with others, etc
	Help sought	Person whom the primary care-givers approached for help—could be immediate family, a relative, neighbour, friend, professional help, traditional healer, or any other
Help-seeking pathway	Reason	What kind of difficulty, improvement not there, continuing difficulties, referral from doctors/other family members
	Source	From where did the family get to know about a particular contact point—referred by a doctor or other people around them or through schools, or was it discovered on their own, e.g., on internet or TV or newspaper, etc
	Contact point	Name, government or a private institution or a non-governmental organisation. Can also include relatives, neighbours, family members, friends, etc
	Location	Facility or home-based
	Advice given	What was the advice given to the family—were they told about what the problem is, any diagnosis given, treatment suggested, was the diagnosis explained, any further referrals given for assessments or treatment, etc
	Service availed	Type of services availed at each institution/organisation—did the family get IQ assessments done or get the hearing assessment done. E.g. Psychological Assessments, blood tests, MRI, etc
	Service provider	The type of service provider, for example, neurologist, paediatrician, psychologist, speech therapist, occupational therapist, etc
	Waiting period	How much time did the family had to wait to get an appointment, after getting an appointment, time lapse between registration and actual meeting with the service provider, etc
	Other remarks	Include information on any other relevant detail that does not fit into the other themes
Autism diagnosis	Contact point for diagnosis	Where and by whom the diagnosis was given—kind of institution and the type of service provider

Finally, results from both the quantitative and qualitative study components were synthesised, triangulated and presented in the discussion.

## Results

### Quantitative Study Participant Characteristics

**Fig. 1:**
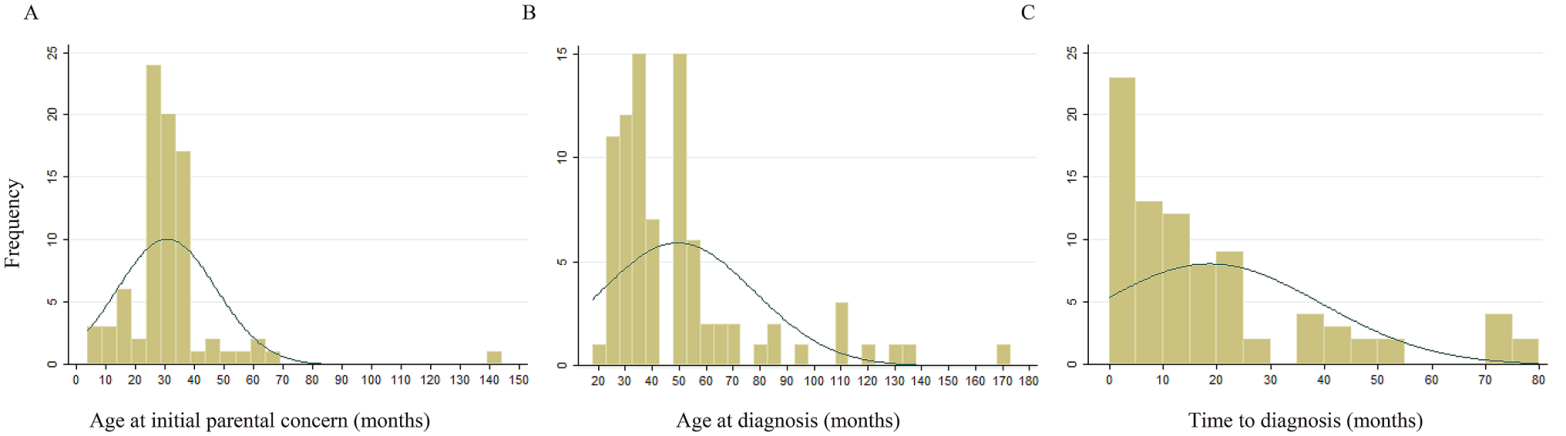
Distribution of **a** age at initial parental concern, **b** age at diagnosis and **c** time to diagnosis in months

The descriptive data of children with autism is summarised in Table 2. Of the 84 children included in the final analysis, 50 (70.24%) were boys. Ten children (12.2%) had comorbid seizures. A quarter of the participating parents (21/84) reported having some knowledge of autism or other developmental disorders prior to their own child’s diagnosis. The age of initial parental concern (Fig. 1a) ranging from 4 months to 12 years (mean = 30.74 ± 16.44 months). The age at autism diagnosis (Fig. 1b) ranged from 18 months to 14.25 years (median = 42 months, IQR = 24 months). The time to diagnosis (Fig. 1c) varied such that there were some children who experienced no delay and others with a duration of 6.5 years between initial parental concern and autism diagnosis (median = 12 months, IQR = 21 months).

**Table 2:** Description of participants in quantitative study component

Characteristic	Total N = 84
Male, n (%)	59 (70.24)
Parental concern^b^	
Social communication, n (%)	55 (68.75)
Restricted repetitive behaviours, n (%)	39 (48.75)
Hyperactivity, n (%)	19 (23.75)
Attention problems, n (%)	14 (17.5)
Behavioural problems, n (%)	13 (16.25)
Language problems, n (%)	55 (68.75)
Reduced sleep, n (%)	3 (3.75)
Seizures, n (%)^a^	10 (12.20)
Prior knowledge of developmental disorders, n (%)	21 (25)
CARS score, mean (sd)	40.76 (4.44)
Age at initial parental concern (months), mean (sd)	30.74 (16.44)
Age at diagnosis (months), median (IQR)	42 (24)
Time to diagnosis (months), median (IQR)	12 (21)

^a^N = 82, ^b^N = 80

### Types of Presenting Concerns

Presenting concerns recognised were categorised into either those pertaining to ‘social communication and interaction’ or ‘restricted and repetitive behaviours’ as per the DSM-V definition of autism (American Psychiatric Association [APA], 2013). Other common parental concerns were found to be (a) hyperactivity, (b) lack of attention, (c) disruptive or challenging behaviours, (d) a lack of receptive and/or expressive language and (e) reduced sleep.

Problems in social communication and an absence of language were reported by 68.75% parents and was the most frequent presenting concerns. Guided by existing autism literature, which highlights differences in the types of symptoms recognized in boys and girls (Halladay et al., 2015; Lockwood Estrin et al., 2020), we analysed whether the types of parental concerns differed by sex (reduced sleep was not included in subsequent analysis due to small numbers). In our sample, the prevalence of the types of presenting concerns was not found to differ between boys and girls except for results suggesting that the absence of language was noticed more often in girls than boys (Fisher's exact = 0.073).

### Factors Impacting Age at Initial Parental Concern, Age at Diagnosis and Time to Diagnosis

We investigated whether the age at parental concern, age at diagnosis and time to diagnosis differed based on types of presenting concerns and prior knowledge. This data was initially analysed separately for boys and girls, however results did not differ by sex and so the results in Table 3 are reported for all children together. While the presence of comorbid seizures did not impact the age at initial parental concern, it took significantly longer for these children to obtain a diagnosis. Sex, the type of parental concern and parents’ prior knowledge of developmental disorders were not found to impact the age of initial parental concern, age at autism diagnosis and time to diagnosis (see Table 3).

**Table 3:** Comparison of age of child at initial parental concern, ASD diagnosis and time to obtain a diagnosis across factors considered in this study

Characteristic (N = 84)	Category	Age at initial parental concern (months)		Age at diagnosis (months)		Time to diagnosis (months)	
		Mean (SD)	t (p value)	Median (IQR)	z (p value)	Median (IQR)	z (p value)
Sex	Male	32.39 (18.40)	− 1.41 (0.16)	42 (24)	− 0.47 (0.64)	12 (20)	− 0.18 (0.86)
	Female	26.84 (10.81)		42 (16)		12 (22)	
Social communication concern^b^	Absent	28.64 (11.54)	− 0.42 (0.68)	48 (30)	0.49 (0.62)	18 (18)	1.34 (0.18)
	Present	29.78 (11.18)		36 (22)		11 (22)	
Restricted repetitive behaviours^b^	Absent	27.20 (9.68)	− 1.85 (0.07)	36 (18)	− 1.12 (0.22)	12 (18)	− 0.02 (0.99)
	Present	31.77 (12.36)		42(28)		12 (34)	
Hyperactivity^b^	Absent	31.37 (17.98)	0.64 (0.52)	42 (22)	0.04 (0.97)	12 (21)	− 0.19 (0.85)
	Present	28.58 (11.02)		42 (24)		12 (21)	
Attention problems^b^	Absent	31.56 (17.89)	1.01 (0.32)	42 (22)	0.96 (0.34)	12 (24)	0.34 (0.73)
	Present	26.64 (6.86)		39 (18)		12 (15)	
Behavioural problems^b^	Absent	28.54 (10.72)	− 1.62 (0.11)	36 (22)	− 1.26 (0.21)	12 (22)	− 0.16 (0.88)
	Present	34 (13.11)		48 (24)		12 (18)	
Language problems^b^	Absent	29.52 (11.85)	0.05 (0.96)	36 (24)	− 0.26 (0.79)	12 (22)	− 0.33 (0.74)
	Present	29.38 (11.05)		42 (19)		12 (20)	
Seizures^a^	Absent	29.39 (11.46)	0.50 (0.96)	36 (19.5)	− 2.75 (0.01)*	12 (19)	− 2.50 (0.01)*
	Present	29.2 (8.70)		63 (36)		37 (36)	
Prior knowledge	Absent	29.81 (10.46)	− 0.88 (0.38)	42 (24)	0.31 (0.76)	12 (22)	− 0.25 (0.80)
	Present	33.52 (28.26)		42 (18)		12 (18)	
CARS score	NA		− 0.16 (0.30)		0.19 (0.08)		0.30 (0.00)*

*Comparisons with p < 0.05

^a^N = 82, ^b^N = 80

### Synthesis of Qualitative Data: A Complex Help-Seeking Pathway

Semi-structured interviews were conducted with parents or caregivers of 20 children with autism and their demographic profile was similar to the quantitative sample (Supplementary Table S1). Six children had diagnosed comorbid conditions of ADHD (4), seizures (2) or Cornelia De Lange Syndrome (1). The mother was the primary respondent in most cases (55%), followed by the father (20%). In four interviews both parents participated equally and the grandparents were respondents in one interview. The majority (72.2%) of the families were nuclear. The following themes arose from within the two areas of the framework analysis and are elaborated below: 1—Initial recognition—Theme 1: Lack of awareness of milestones of child development, Theme 2: Recognition of behavioural or developmental concerns and Theme 3: Disempowerment of mothers and lack of social support; 2—Help-seeking pathway—Theme 1: Initial contact with the health system. Theme 2: Variability in diagnosis practices amongst child development specialists and Theme 3: A non-linear pathway to diagnosis.

#### Initial Recognition

This area of inquiry describes three themes: the difficulties noticed by caregivers, help sought and advice given during initial recognition of developmental or behavioural concerns in a child.

##### Theme 1: Lack of Awareness of Milestones of Child Development:

A lack of awareness in the community of healthy neurodevelopment during early childhood emerged as a common theme across the interviews.

“We felt that.... He is physically fine. No one can say by looking at him that he might have any problem. But lack of understanding and speech matters a lot. We understood it then.”—mother

This is also reflected in a wide age-range at which parents initially noticed developmental concerns in their child with some parents beginning to seek help only when the child was 4–5-years old. Delayed attainment of developmental milestones in young children was found to be widely accepted. Particularly striking was the oft-repeated belief that boys speak later than girls and that children can start speaking at any age, including as late as 6–9 years. Examples of family members, most often the father, who had apparently also displayed delays in development in childhood, served as reassurances to the concerned parent.

“They said that father also didn’t speak till the age of five. [He went on to do] engineering, was successful, … [achieved] first division, no tension.”—mother

“She [mother in law] gave a general opinion that it happens—‘it is normal that some children speak late. A child in our distant relation also started speaking at the age of 7–8 years but is now aeronautical engineer.... is very intelligent’.”—mother

Behavioural concerns were dismissed as being either a sign of the uniqueness of a child or harmless childish behaviours like irritability, naughtiness and bad habits.

“You are worried unnecessarily. Everything will be fine. Some children are unique"—father’s response to mother’s concerns

“Sometimes she also created it. If she is irritated. (referring to autistic symptoms that the child shows)… maybe iron deficiency also causes irritability.”—mother

##### Theme 2: Recognition of Behavioural or Developmental Concerns:

Parents most often noticed the presence of developmental or behavioural concerns in their children but in some cases these were also brought to their notice by extended family members and visiting relatives. In the case of older children who were enrolled in preschools, teachers too expressed concerns about the child’s development or behaviour and recommended that parents consult a doctor. Apart from their spouse, parents with concerns about their child often reached out to their immediate family members for advice. Advice was also sought from extended family, neighbours and in a few cases, Homeopathy and Ayurveda practitioners. Upon receiving conflicting advice from these people, parents reported experiencing a sense of confusion about how to proceed. Overwhelmingly though, we found that parents were advised to ‘wait and watch’ their child with the belief that they would ‘catch-up’ in their development (elaborated later). Only in a few cases were parents immediately advised to visit a doctor.

Parents mostly heard of autism for the first time when their child received the diagnosis. Interestingly, some parents reported that despite having some knowledge of autism, in one case through a popular television programme, they did not feel that the concerns they noticed in their own child could be attributed to, or were severe enough, to match their own prior perception of the disorder. Parents also reported a lack of awareness of diagnostic and remedial services, like child development centres, for young children with concerns in their development and expressed that this translated into a feeling of helplessness.

##### Theme 3: Disempowerment of Mothers and Lack of Social Support:

Another common theme that emerged from the interviews with mothers was a sense of their disempowerment. Interviewed mothers echoed the feeling that women were not empowered enough to act on their concerns about their child without the support of their husbands and family members due to a lack of agency and involvement in decision making about their children’s health. An example was a mother who was a practicing dentist before her child was born, and noticed delayed milestones when her child was as young as 3-months of age but was unable to consult a child development specialist till the child was over 3-years old due to the lack of supportive family members.

I knew from the beginning that child has some problem but it took me so long to know diagnosis. It was because no one supported me for the examination. It was the problem.—mother

No one in the society pays attention. Even now if I tell them to do something.... though I am educated enough to take my own decisions, I can’t.—mother

Mothers also reported that being the sole primary caretaker of multiple children also resulted in their attention being diverted from the autistic child which served as a constraint in timely help-seeking.

#### Help-Seeking Pathway

This topic describes families’ experience as they interacted with the health system to obtain an autism diagnosis for their child.

##### Theme 1: Initial Contact with the Health System:

The first point of contact for parents within the health system was most commonly a general physician or a paediatrician, sometimes known informally to the family, or who was already being visited for routine immunizations or other reasons in their child. Often, the advice to parents to ‘wait and watch’ the child was received from these health professionals as well, which prolonged the pathway to diagnosis.

Actually, my friend is also a paediatrician but she also said to me, ‘It’s nothing. Some children are different’. This also pulled my steps back.—mother

Often concerns about physical conditions were given precedence over autism-related symptoms and in one case, the lack of any concerns about the child’s physical health resulted in a parent being admonished by the doctor.

When autism-related symptoms were given due importance, since these were predominantly social-communication difficulties and lack of language, the first pathway of referral was for audiometric assessments by otolaryngologists. Blood tests, Magnetic Resonance Imaging (MRI) and electroencephalography (EEG) were also found to be commonly recommended secondary investigations, particularly when children presented with comorbid seizures.

##### Theme 2: Variability in Diagnosis Practices Amongst Child Development Specialists:

Diagnosis practices differed amongst child developmental specialists, such that the age at which they were comfortable to diagnose children with autism was varied. In two cases, parents of children aged 2.5 and 3 years old were told that while there seemed to be developmental concerns with their child, a diagnosis could not be given till the child was older and these children finally received an autism diagnosis at 3 and 5-years age respectively. In contrast to this, one child was diagnosed at 1.25-years in another health facility. Parents of diagnosed children were referred to recommended interventions such as speech, occupational or physical therapy and many times these were recommended even before a formal diagnosis of autism was given.

##### Theme 3: A Non-linear Pathway to Diagnosis:

However, even once their child received an autism diagnosis and was undergoing intervention, some parents continued in the help-seeking pathway by consulting multiple health professionals for further confirmation or therapies—in one case 14 health facilities were visited by a family. This sense of confusion is evident in a mother’s own words.

I don’t know. I was confused that time and still am. I have noticed improvement in the child but still have confusion somewhere in my mind—whether it is autism or something else.—mother

In some cases, children were recommended medical or surgical interventions, including hearing aids, tongue-tie division and medications, particularly children who presented with symptoms of ADHD. Families that underwent these interventions would re-enter the help-seeking pathway when concerns about their child’s development persisted and new symptoms emerged as they grew older. Finally, parents also mentioned being faced with navigating an overburdened health system, an example of which was a parent who mentioned having to wait for 6 months for an appointment in a reputed tertiary hospital.

## Discussion

**Fig. 2:**
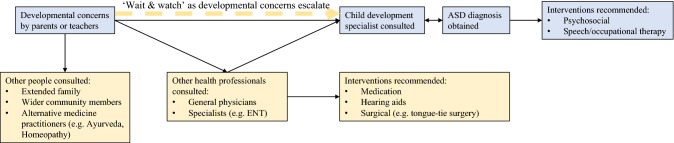
Help-seeking pathway followed by families to obtain an ASD diagnosis

In this study, we have used mixed methods to explore the barriers to obtaining an autism diagnosis in urban north Indian children. We investigated the impact of types of presenting concerns and prior knowledge of developmental disorders on the age of the child at initial parental concern and autism diagnosis. We have complemented this quantitative exploration by using a qualitative approach to identify contextual family, societal and health-system related factors that impede the journey between parental recognition of developmental or behavioural concerns and an autism diagnosis by a child developmental specialist. Triangulating results from across these approaches has revealed the help-seeking pathway followed by parents of children with autism to be a complex and arduous one. The pathway, beginning with recognition of symptoms by parents and teachers, followed by initial contact with the health system and finally resulting in a diagnosis by child development specialists, is summarised in Fig. 2.

On average, children were 2-years old when parents or teachers first noticed developmental or behavioural concerns and 4-years old when diagnosed with autism. This finding replicates recent reports from India and demonstrates a consistency across cities in the north and south of the country and also types of facilities like special schools and tertiary hospitals from which participants were recruited (Kommu et al., 2017; Mahapatra et al., 2019; Preeti et al., 2017). Our qualitative interviews revealed the hesitance of some child developmental specialists to diagnose 2–3-year old children with autism. Similar hesitancies have been reported in other countries, including both LAMIC and HIC, like Nepal (Heys et al., 2017; Shrestha & Shrestha, 2014; Shrestha et al., 2019) and USA (Zuckerman et al., 2017) highlighting that efforts made to demonstrate a stable autism diagnosis at younger ages (Guthrie et al., 2013; Lord et al., 2006; Ozonoff et al., 2015) are yet to translate consistently into clinical practice. Additionally, comorbidities are known to overshadow autism-specific parental concerns and delay autism diagnosis (Daley, 2004; Daniels & Mandell, 2014). In our sample too, children with comorbid seizures received an autism diagnosis later than those without seizures.

We demonstrate that impairments in social communication and the absence of language constitute the most frequent presenting concerns. Similar results have been obtained by other studies from India (Daley, 2004; Mahapatra et al., 2019; Preeti et al., 2017) and Nepal (Shrestha & Shrestha, 2014). However, there is conflicting evidence on differences in presenting concerns based on sex of the child (Lockwood Estrin et al., 2020), and we found some indication of lack of language being a presenting concern more often for girls than boys. This might relate to the theme that emerged from in-depth interviews that there is a widespread belief that boys speak later than girls. Some other examples of how cultural beliefs influence the recognition of autism symptoms are the lack of emphasis on eye contact in Asian cultures (Zhang et al., 2006) and pretend play in multiple LMICs (Edwards, 2000). These cultural nuances, along with non-biological explanatory models of autism, contribute to delayed help-seeking (Leeuw et al., 2020).

Parents relied heavily on advice from family and wider community members, who mostly dismissed parents’ concerns thereby inadvertently impeding their efforts to obtain an autism diagnosis. We also found that mothers with unsupportive partners and extended family members felt disempowered to act on their concerns about their child’s development. A recently published meta-synthesis of the experience of parents of Asian children with autism has also shown this disempowerment of mothers to be a barrier to help-seeking, particularly in Middle-Eastern cultures (Shorey et al., 2019). Interestingly, we found that preschool teachers were often the first to raise concerns with parents, signposting them to doctors. The potential of pre-school teachers as facilitators in the pathway to autism diagnosis needs to be recognised and built upon (Drusch, 2015).

We found that majority of the parents were unaware of autism or any other developmental disorder prior to their own child’s diagnosis. A similar lack of awareness of autism has been reported from other communities from LMICs such as Somali families living in the UK (Hussein et al., 2019) and families in Pakistan (Minhas et al., 2015). Interestingly though, in this study, even parents that did claim to have prior knowledge of any developmental disorders did not have a shorter pathway to diagnosis than those that didn’t. Our qualitative data revealed that this might be because parents did not ascribe their concerns of their own child’s development or behaviours with their *perception or understanding* of autism. Furthermore, an acute lack of awareness of the importance of age-appropriate attainment of developmental milestones was apparent in the parents who were interviewed and a concerted effort needs to be made to emphasise these amongst the general population. High quality resources developed to help educate parents on child development already exist in multiple forms in most countries, for instance the Mother and Child Protection Card in India (*Home:: National Health Mission*, n.d.). These include emphasising the importance of social communication milestones like making eye contact, responding to their name and smiling socially. These need to be used more effectively by both parents and lay health care workers, and could be localised to include direct information about referral pathways for children not meeting developmental milestones. This would help raise awareness of available early childhood development health services, such as like child development clinics, and thereby avoid the need for parents to visit multiple health professionals and facilities. This, in turn, would help shorten the pathway to an autism diagnosis.

The effort to increase awareness of development during early childhood needs to include a family’s first point of contact within the health system such as general physicians and vaccination nurses in government and private health facilities. Signs of autism were often missed by these health professionals too, and parents with concerns about their child’s development were dismissed rather than being referred to early childhood development specialists for diagnostic evaluations and interventions. A recent quantitative analysis of the profiles of children with autism who consulted a tertiary hospital in Bangalore has demonstrated a delay of over 1.5-years between parents first consulting a health professional for developmental concerns in their child, and receiving an autism intervention, and highlighted the need for health professionals to facilitate referrals to early interventions (Preeti et al., 2017). Similar findings have been reported in other LAMIC like Pakistan (Minhas et al., 2015), Nepal (Shrestha et al., 2019) and in HIC like the US (Zuckerman et al., 2015a, 2015b). Apart from prolonging the delay in autism diagnosis, the implications of this lack of awareness of autism amongst doctors in low resource settings are compounded as it leads to parents allocating scarce resources on relatively expensive interventions like hearing aids and tongue-tie surgeries that are unnecessary for core autism impairments (see Fig. 2).

In addition to increasing awareness of autism amongst these health professionals, they need to be equipped with screening tools that can flag signs of autism. The Modified Checklist for Autism in Toddlers Revised with Follow-up (M-CHAT-R/F); Pictorial Autism Assessment Schedule (PAAS), Three-Item Direct Observation Screen (TIDOS) have been identified by a recent systematic review as three tools that have the potential to be used in LAMIC (Marlow et al., 2019). Harnessing the potential of scalable digital tools designed to capture and analyse child behaviour without depending on detailed observations by trained specialists or on parent-report (Chakrabarti, 2019; Durkin et al., 2015), will allow us to enlist the large cadre of non-specialist healthcare workers that already work in the maternal and child health governmental department in most LAMIC; for instance, the Accredited Social Health Activist (ASHA) workers in India. In parallel to the effort to strengthen the pathways to autism diagnosis, there needs to be a concerted effort towards ensuring the provision of accessible and effective interventions that are known to be able to improve social-communication and adaptive behavioural outcomes for children with autism (Green & Garg, 2018).

We acknowledge the limitations of this study. Participants were recruited from premier tertiary hospitals in one of the largest cities in India and thus our findings of contextual barriers that impede the help-seeking pathway are unlikely to be representative of the general population, particularly those residing in rural settings, or to other LAMIC. For instance, in our study, while we did encounter families that sought help from practitioners of alternative medicine like Homeopathy or Ayurveda, faith healers were not consulted. This is contrast to other reports from other countries, including India (Divan et al., 2012; Höfer et al., 2016). Another limitation of the study is that the data about age at initial parental concern and age at autism diagnosis have been collected retrospectively, and are subject to recall bias. Additionally, we do not have any information on the socioeconomic status of our participants, limiting our ability to examine it as a determinant of delayed diagnosis in this study.

The combination of increased awareness and scalable screening tools will enable flagging of children at risk for autism and their timely referral to child development specialists, while at the same time reducing the burden on this scarce resource. It will also allow us to move beyond using growth measures like height and weight as proxy for developmental attainment in early childhood and truly achieve developmental monitoring to ensure that every child with a diagnosis of autism thrives (Divan et al., 2021).

## Supplementary Information

Below is the link to the electronic supplementary material.

Supplementary file1 (DOCX 13 kb)
