# Supplementary material for: “I was Confused … and Still am” Barriers Impacting the Help-Seeking Pathway for an Autism Diagnosis in Urban North India: A Mixed Methods Study
Source: J Autism Dev Disord. 2021 May 20;52(4):1778–88. doi: 10.1007/s10803-021-05047-z (PMC8938390; doi:10.1007/s10803-021-05047-z)
Supplement: Supplementary file 1 — Supplementary file1 (DOCX 13 kb) [file 10803_2021_5047_MOESM1_ESM.docx]

*Supplementary table S1.* Description of qualitative study participants

| Characteristic |  |
| --- | --- |
| Male, n (%) | 14 (70%) |
| Age of child at interview | 89.26 (29.62) |
| Age of child at initial parental concern (months), mean (sd) | 21.05 (11.84) |
| Age of child at diagnosis (months), mean (sd) | 53.49 (20.50) |
| Time taken to obtain a diagnosis (months), mean (sd) | 31.61 (22.33) |
| Comorbidities, n (%)  ADHD  Seizures  Cornelia De Lange Syndrome | 4 (20%)  2 (10%)  1 (5%) |
| Nuclear family, n (%)* | 14 (72.2%) |
| Primary respondent, n (%)  Mother  Father  Both parents  Grandparents | 11 (55%)  4 (20%)  4 (20%)  1 (5%) |

*Missing data for 2 participants
